# Association of Virtual Care Expansion With Environmental Sustainability and Reduced Patient Costs During the COVID-19 Pandemic in Ontario, Canada

**DOI:** 10.1001/jamanetworkopen.2022.37545

**Published:** 2022-10-20

**Authors:** Blayne Welk, Eric McArthur, Alexandra P. Zorzi

**Affiliations:** 1 Department of Surgery and Epidemiology and Biostatistics, Western University, London, Ontario, Canada; 2 ICES, Ontario, Canada; 3 Department of Pediatrics, Western University, London, Ontario, Canada

## Abstract

**Importance:**

There is a growing focus on environmental sustainability in health care.

**Objective:**

To estimate the environmental and patient-level financial benefits associated with the widespread adoption of virtual care during the COVID-19 pandemic.

**Design, Setting, and Participants:**

This population-based cross-sectional study obtained data from linked administrative databases in the universal health care system of Ontario, Canada, from March 2020 to December 2021. Participants included all people with a physician claim for at least 1 episode of virtual care.

**Exposures:**

Patients were stratified by age, socioeconomic status quintiles, Charlson Comorbidity Index, and area of residence (rural or urban).

**Main Outcomes and Measures:**

The primary outcomes were total travel distance and estimated travel-related carbon dioxide emissions avoided owing to virtual care visits. Different model assumptions were used to account for electric and hybrid vehicles and public transit use. The secondary outcomes were estimated patient costs (gasoline, parking, or public transit expenses) avoided.

**Results:**

During the 22-month study period, 10 146 843 patients (mean [SD] age, 44.1 [23.1] years; 5 536 611 women [54.6%]) had 63 758 914 physician virtual care visits. These visits were associated with avoidance of 3.2 billion km of travel distance and between 545 and 658 million kg of carbon dioxide emissions. Patients avoided an estimated total of $569 to $733 million (Canadian [US $465-$599 million]) in parking, public transit, and gasoline costs. Carbon dioxide emission avoidance and patient cost savings were more apparent in patients living in rural areas, those with higher comorbidity, and those who were older than 65 years.

**Conclusions and Relevance:**

Results of this study suggest that virtual care was associated with a large amount of carbon dioxide emissions avoided owing to reduced patient travel and with millions of dollars saved in parking, gasoline, or public transit costs. These benefits are likely to continue as virtual care is maintained as part of the health care system.

## Key Points

**Question:**

How much carbon dioxide emissions and patient travel–related costs were reduced by the expansion of virtual care during the initial 22 months of the COVID-19 pandemic in Ontario, Canada?

**Findings:**

In this cross-sectional study of more than 10 million patients and 63 million virtual care visits, virtual care was associated with avoidance of 3.2 billion km of patient travel, 545 to 658 million kg of carbon dioxide emissions, and $569 to $733 million (Canadian [US $465-$599 million]) in expenses for gasoline, parking, or public transit.

**Meaning:**

Findings of this study suggest that these environmental and financial benefits may continue as virtual care is maintained as part of the health care system.

## Teaser

This cross-sectional study examines the association of virtual physician visits with reducing carbon dioxide emissions and transportation–related patient expenses during the beginning of the COVID-19 pandemic.

## Introduction

The COVID-19 pandemic necessitated the rapid expansion of virtual care in many countries. Early in the pandemic, virtual care was a means of maintaining nonemergent patient care during stay-at-home orders; after the removal of these public health restrictions, it has continued to be an integral part of the health care system.^1,2^ Estimates from the US suggest that use of virtual care increased 38-fold compared with prepandemic levels and may in the future account for a quarter of a trillion dollars of health care spending.^2^ In Canada, virtual care was also broadly adopted early in the pandemic and has continued despite lessening COVID-19 restrictions.^3^ In Ontario (Canada’s largest province), 70% to 80% of all physician visits were virtual during the first 3 months of the pandemic (April-June 2020) and then stabilized at 50% to 60% of visits.^4^ Before the pandemic, virtual care reimbursement was restricted to a single hospital-based videoconferencing service and made up less than 2% of patient visits.^5^ The rapid uptake of virtual care was precipitated by physician and patient willingness to provide and use virtual options as well as rapid changes to regulatory and compensation rules for virtual care.^1^ With the widespread adoption of virtual care, the total combined number of in-person and virtual care episodes during the pandemic was stable for more than 90% of physicians in Ontario, Canada,^6^ suggesting that virtual care replaced rather than supplemented most episodes of in-person care.

There has been substantial research into the safety and validity of virtual care,^7^ and most patients have favorable views of using virtual care (even more so during the pandemic).^8^ However, there has been little discussion on the potential environmental and fiscal benefits that society may derive from virtual care. The objective of this study was to estimate the environmental and patient-level financial benefits associated with the widespread adoption of virtual care during the COVID-19 pandemic.

## Methods

### Design, Setting, and Data Sources

We conducted a cross-sectional study using linked administrative databases from Ontario, Canada, with a population of approximately 14.7 million, from March 2020 to December 2021. All residents of Ontario use a publicly funded health care system. The use of data in this study was authorized under section 45 of the Ontario Personal Health Information Protection Act, which did not require the study to be reviewed by a research ethics board and did not require informed consent from participants. We followed the Strengthening the Reporting of Observational Studies in Epidemiology (STROBE) reporting guideline.

We retrieved data from 4 ICES databases for this study. First, we used the Ontario Health Insurance Plan database, which is a record of all physician claims for fee-for-service compensation. These payment records have good accuracy and completeness.^9^ Second, we used the Registered Persons Database, which provides basic demographic information, such as age and residential postal code, for all Ontario residents. Third, we used the ICES Physician Database, which includes demographic information for all physicians and the postal code of their practice location. Fourth, we used the Canadian Institute for Health Information Discharge Abstract Database, which is populated by trained abstractors and includes demographic, diagnostic, and procedural variables for all hospital admissions in Ontario. Previous quality assessments have demonstrated that the Discharge Abstract Database has good accuracy and completeness compared with reabstracted medical records.^10^ These 4 data sets were linked using unique encoded identifiers and were analyzed at ICES.

### Study Cohort

We included all patients in Ontario with at least 1 virtual care encounter (Ontario Health Insurance Plan fee code K080, K081, K082, or K083) between March 14, 2020, and December 31, 2021. These billing codes were newly introduced in March 2020 to reimburse physicians for virtual care services at rates approximately equal to in-person care. Virtual care could be delivered by a telephone or video call. We excluded patients with invalid data elements (invalid unique identification numbers, non-Ontario residency, and death before the date of the virtual care claim) or a missing postal code. We excluded a patient visit if the physician providing the virtual care did not have a valid identification number or had a missing practice postal code. We allowed a maximum of 1 virtual care visit per patient per physician per day. Race and ethnicity data were not collected because they were not available in the administrative data sets.

### Outcomes

The primary outcomes were the total travel distance avoided and the travel-related carbon dioxide (CO_2_) emissions avoided for the virtual care visits during the study period. The total travel distance avoided was calculated by converting the postal code of the patient’s area of residence (from the Registered Persons Database) and the physician’s place of practice (from the ICES Physician Database) into longitude and latitude coordinates. The distance between these 2 points was then calculated in kilometers using the orthodromic distance (shortest path between points on a sphere) and doubled to account for travel to and from the physician’s office. The travel-related carbon dioxide emissions avoided were estimated as the product of the total travel distance avoided and the CO_2_ emissions for the average passenger vehicle in Canada (206 g CO_2_/km).^11^ A second calculation was performed to account for the proportion of electric vehicles in Ontario (based on approximately 1.8% of passenger vehicles being fully electric and 3.0% being hybrid).^12,13^ For this calculation, fully electric cars were estimated to generate 7.6 g CO_2_/km (due to the electricity production used to charge the vehicle), and hybrid vehicles were estimated to produce 95 g CO_2_/km.^14^ Because there are no available estimates on the proportion of people who use public transit to attend medical visits in Ontario, we estimated CO_2_ emissions on the basis of both 10% and 20% of people using public transit^15^ and estimated 60 g CO_2_/km for this group.^16^

The secondary outcomes were patient expenses avoided due to the virtual care visit replacing an in-person visit. First, parking or public transit costs were estimated at $5 for family physician visits and $10 for specialist visits; for the approximately 10% to 20% of people who may use public transit for commuting,^15^ we estimated that the cost of public transit would be equivalent to the cost of parking. We also calculated parking or public transit costs with a more conservative model by taking into account regional differences in parking costs in Ontario.^17^ In this model, parking costs were estimated differently for physicians practicing in rural areas: the cost of family physician visit parking was 0, and specialist visit parking was $5. In this model, we also assumed 20% of people used public transit without any additional costs. Second, gasoline costs were estimated with this formula: mean fuel efficiency of a typical passenger vehicle in Canada (0.087 L gasoline/km)^11^ × total kilometers traveled × mean cost per liter of regular gasoline in Ontario that month.^18^ A second model was calculated using the same assumptions for travel-related CO_2_ emissions (accounting for electric and hybrid vehicles and public transit use at 10% and 20%). The cost of charging an electric vehicle was estimated at 2.1 cents/km, the mean fuel efficiency of a hybrid vehicle was estimated at 4.3 L gasoline/100 km, and the estimated proportion of public transit kilometers was excluded from the gasoline costs.^11^ All costs were reported as Canadian dollars (CAD), with US dollar conversion based on the mean exchange rate in 2021 of CAD $1.2538 for US $1.

### Comparator Groups

We evaluated the number of virtual care visits and the travel distance avoided owing to virtual care visits in 4 subsets. We stratified the patients by age (<18, 18-65, and >65 years, which were obtained from the Registered Persons Database) and socioeconomic status (SES) quintiles. The SES quintiles were derived from the median household income within the person’s neighborhood (using postal code data from the Registered Persons Database). We used the Charlson Comorbidity Index (CCI) score of less than 2 (indicating low comorbidity) and 2 or higher (indicating high comorbidity), calculated with the past 2 years of records in the Discharge Abstract Database.^19^ Moreover, we identified people living in a rural area by their rurality score, which is based on community population density and distance to basic and advanced medical referral centers.^20^

### Statistical Analysis

Data analysis was conducted from May 15 to August 30, 2022, using SAS, version 9.4 (SAS Institute Inc). Where appropriate, the data were summarized as a mean (SD) or a median (IQR), and we also reported the 95th and 99th percentiles of the number of virtual visits and total kilometers avoided. Density plots were created with R, version 4.2.1 (R Foundation for Statistical Computing) to visualize the distributions of the number of virtual care visits and the total travel distance avoided across the comparator groups. There were no missing data. A 2-tailed *P* < .05 was considered to be significant.

## Results

**Figure 1.**
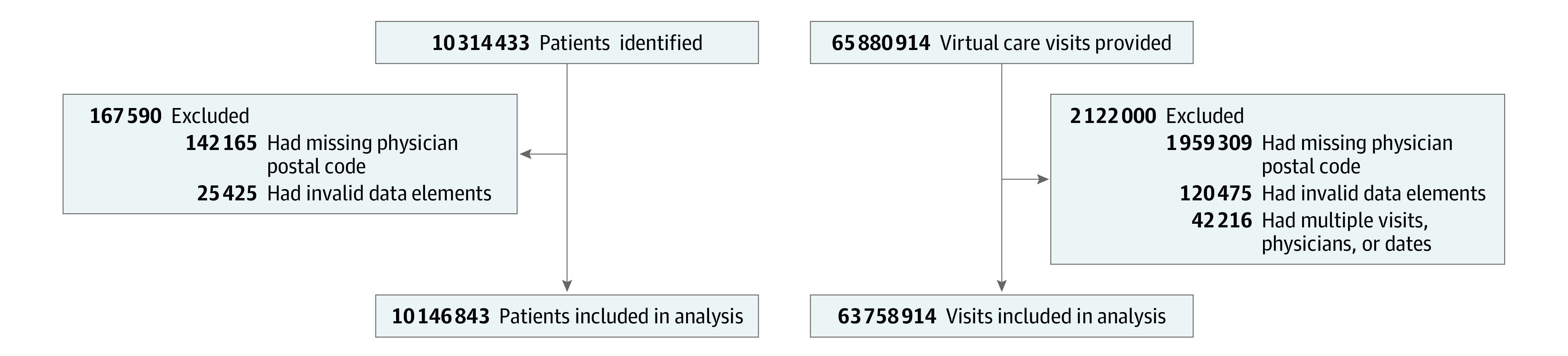
Flowchart of the Study Cohort

**Figure 2.**
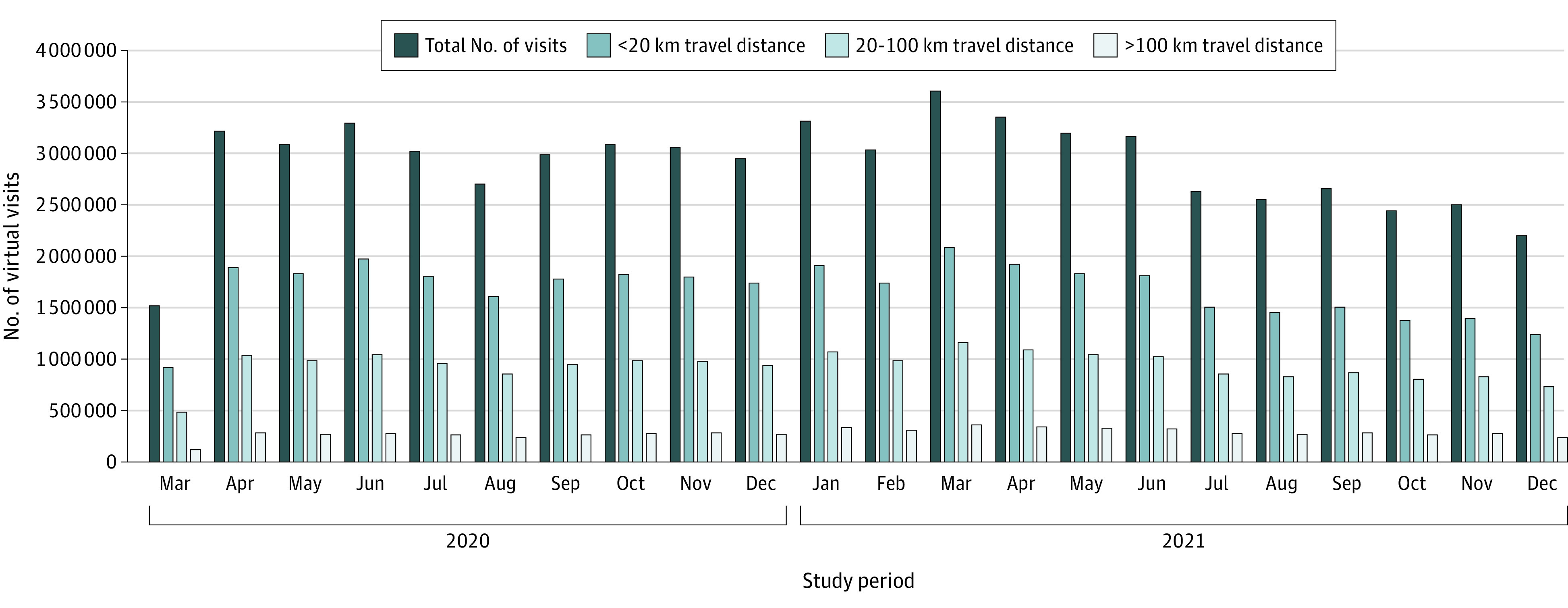
Number of Virtual Care Visits Stratified by Round-Trip Travel Distance Between Patient Area of Residence and Physician Place of Practice

We identified 10 146 843 patients (mean [SD] age, 44.1 [23.1] years; 5 536 611 women [54.6%]) and 4 610 232 men [45.4%]) after applying the exclusion criteria (Figure 1), and these patients had 63 758 914 physician virtual care visits. This total represented a mean of 6.3 virtual visits per person during the study period or 3.5 virtual visits per person per year. Virtual care was associated with a total of 3.2 billion km of travel distance avoided (mean [SD], 50.3 [151.2] km round trip/virtual care visit) over the initial 22 months of the COVID-19 pandemic (Figure 2). We found that 658 million kg of vehicle travel–related CO_2_ emissions were avoided (mean [SD], 10.4 [31.3] kg CO_2_/virtual visit). In the secondary models that accounted for electric or hybrid vehicles and public transit, the travel-related CO_2_ emissions avoided were estimated at 589 million kg of CO_2_ (assuming 10% use of public transit) and 545 million kg of CO_2_ (assuming 20% use of public transit). These secondary models equated to a mean (SD) of 9.3 (30.0) kg CO_2_ emissions avoided per virtual visit for 10% use of public transit and 8.4 (25.3) kg CO_2_ emissions avoided per virtual visit for 20% use of public transit.

For the secondary outcome of patient costs, we estimated savings of CAD $569 million (US $465 million) to CAD $733 million (US $599 million) during the 22-month study period. Specifically, patients avoided CAD $409 million (US $326 million) in parking or public transit fees. In the more conservative secondary model that accounted for reduced parking costs in rural locations and no incremental costs for an estimated 20% of people using public transit, this cost was estimated at CAD $319 million (US $254 million). Patients avoided CAD $324 million (US $258 million) in gasoline costs. In the secondary model, after accounting for electric and hybrid vehicles and public transit, the cost of gasoline or vehicle charging avoided was estimated at CAD $282 million (US $225 million) if 10% of the population used public transit and CAD $250 million (US $199 million) if 20% of the population used public transit.

**Figure 3.**
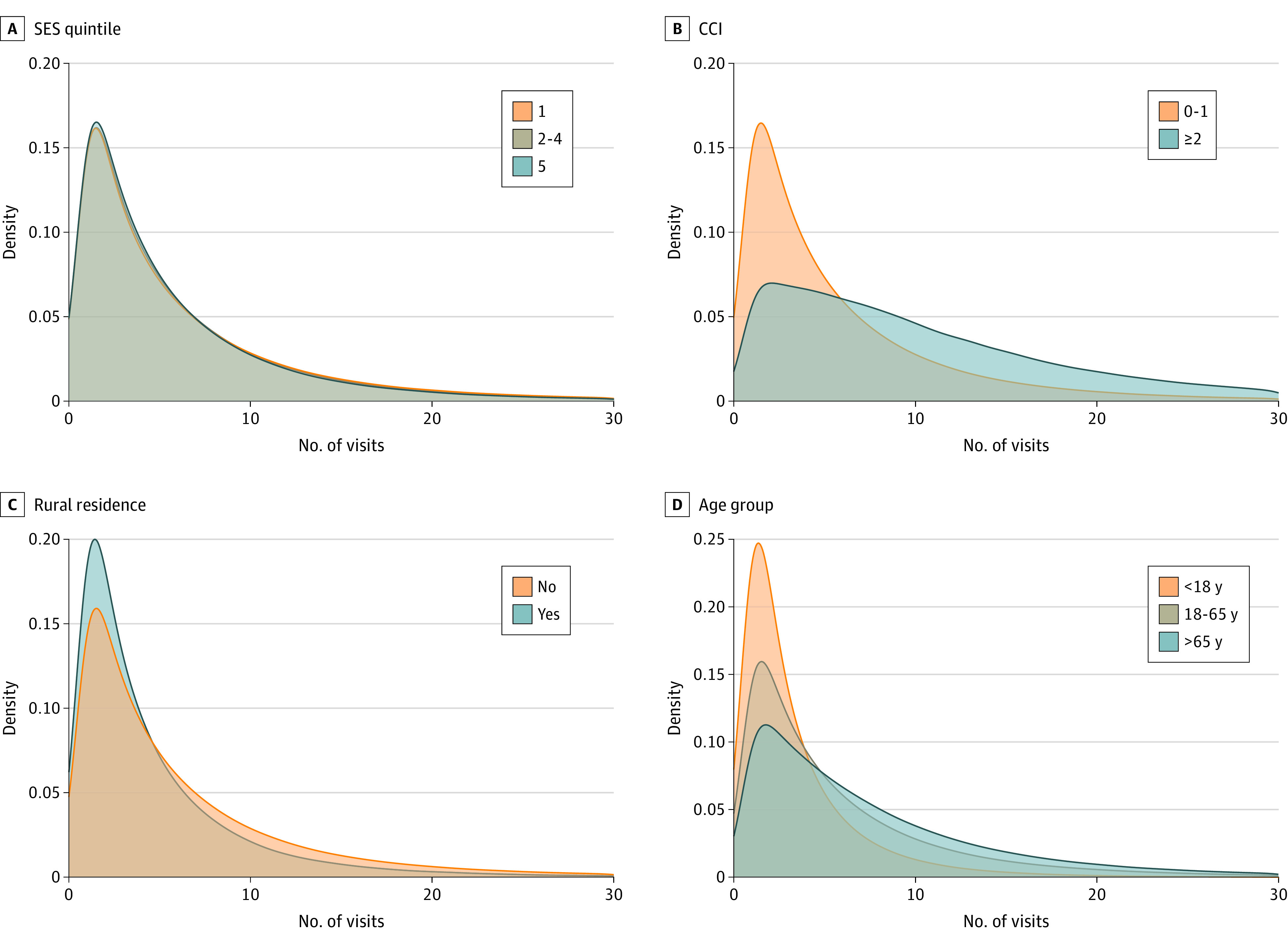
Density Plots of the Number of Visits per Patient Stratified by Socioeconomic Status (SES) Quintile, Charlson Comorbidity Index (CCI) Score, Rural Residence, and Age SES quintile 1 indicates lowest SES and quintile 5 indicates highest SES. CCI score of less than 2 indicates low comorbidity; greater than or equal to 2 indicates high comorbidity.

**Figure 4.**
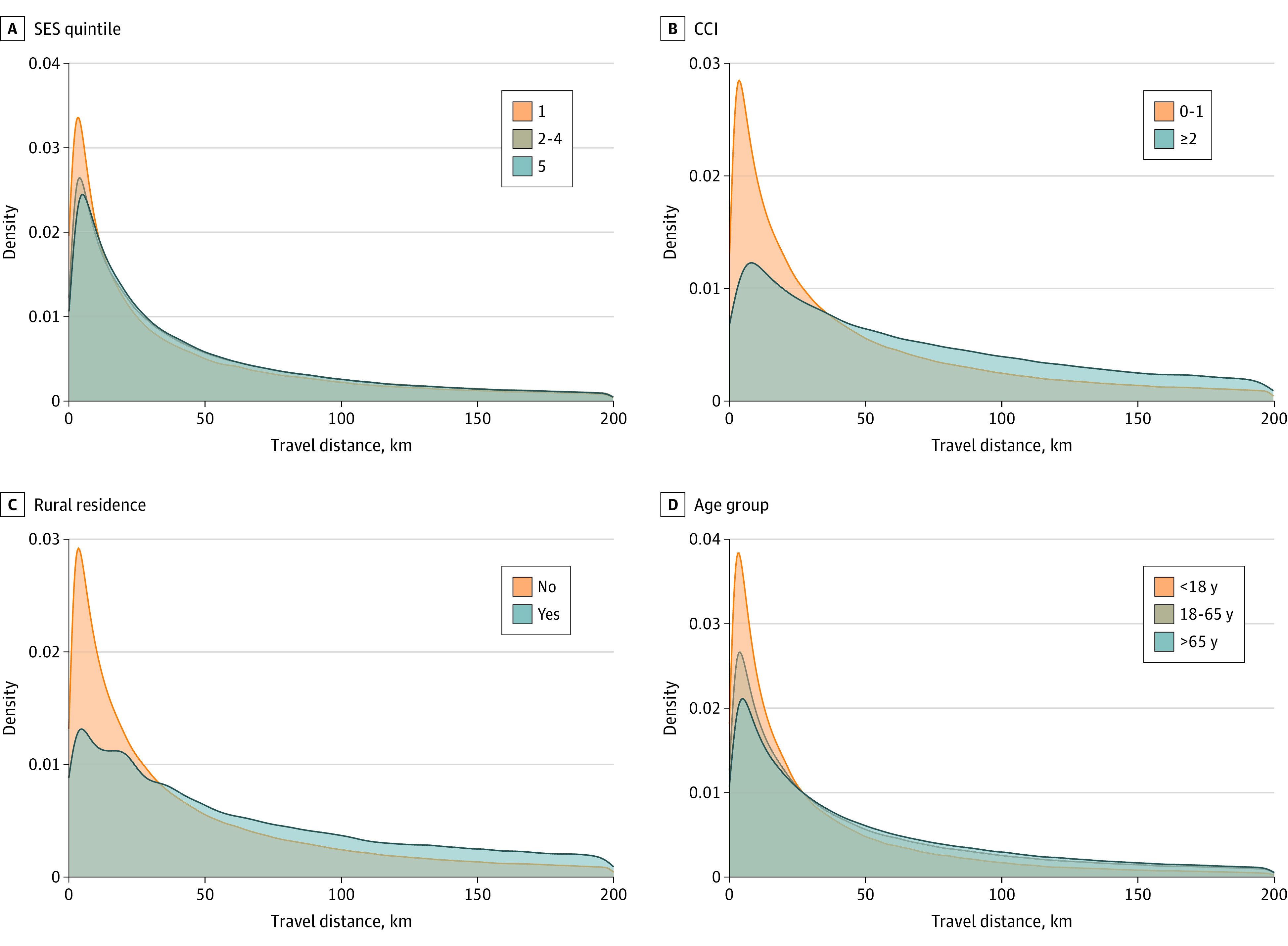
Density Plots of the Total Kilometers of Travel Distance Avoided per Patient Stratified by Socioeconomic Status (SES) Quintile, Charlson Comorbidity Index (CCI) Score, Rural Residence, and Age SES quintile 1 indicates lowest SES and quintile 5 indicates highest SES. CCI score of less than 2 indicates low comorbidity; greater than or equal to 2 indicates high comorbidity.

The number of visits and number of kilometers of potential travel distance avoided per person are shown in the Table and stratified by age, SES quintile, CCI, and area of residence (rural or urban). The distribution of the number of virtual care visits by group (age, SES quintile, CCI score, and rural residence) is shown in Figure 3. There was a more pronounced increase in the proportion of patients with a large number of virtual care visits among those with a CCI score of 2 or higher and those older than 65 years. The distribution of kilometers of travel distance avoided among these groups is shown in Figure 4. However, a visibly larger proportion of patients had more kilometers of travel distance avoided among those with a CCI score of 2 or higher (due to a higher number of virtual care visits) and among those living in rural areas (due to a longer distance between their area of residence and their physician’s place of practice).

**Table.** Number of Visits and Number of Kilometers of Travel Distance Avoided per Individual, by SES Quintile, CCI Level, and Area of Residence

Factor	No. of people	No. of visits				Travel distance avoided due to virtual care visits, km			
		Total	Median per person (IQR)	95th Percentile	99th Percentile	Total	Median per person (IQR)	95th Percentile	99th Percentile
Overall	10 126 462	63 556 271	4 (2-8)	20	36	3 197 099 600	82 (25-262)	1354	3769
Age, y									
<18	1 573 365	5 466 172	2 (1-4)	10	18	247 470 808	44 (15-129)	653	1867
18-65	6 368 561	40 144 091	4 (2-8)	20	36	2 195 203 342	89 (27-286)	1482	4088
>65	2 184 536	17 946 008	6 (3-11)	24	40	754 425 450	107 (34-314)	1405	3698
SES quintile									
Lowest	1 874 158	12 308 274	4 (2-8)	21	38	610 671 876	69 (19-245)	1409	4010
Highest	2 114 484	12 722 608	4 (2-8)	19	34	638 797 114	85 (28-256)	1218	3394
CCI score^a^									
Low (CCI <2)	9 904 178	60 891 564	4 (2-8)	20	35	3 065 064 310	81 (25-255)	1327	3670
High (CCI ≥2)	222 284	2 664 707	9 (4-16)	33	53	132 035 290	214 (71-597)	2317	5723
Area of residence									
Urban	9 242 412	59 402 929	4 (2-8)	20	36	2 631 966 192	76 (24-235)	1191	3432
Rural	879 570	4 125 545	3 (1-6)	14	26	563 032 828	215 (67-640)	2577	6109

Abbreviations: CCI, Charlson Comorbidity Index; SES, socioeconomic status.

^a^ Measured by CCI, with a score of less than 2 indicating low comorbidity and greater than or equal to 2 indicating high comorbidity.

## Discussion

We estimated the environmental benefits and selected patient-level cost savings associated with the widespread adoption of virtual care during the COVID-19 pandemic in Ontario, Canada. The travel distance avoided was estimated at 3.2 billion km, which is equivalent to driving the circumference of the earth 80 000 times or traveling to and from the moon 4158 times. This distance was associated with an estimated 545 to 658 million kg of CO_2_ emissions avoided over 22 months, which represented approximately 0.2% of the total annual CO_2_ emissions (150 megatons) from Ontario.^21^

The per-visit CO_2_ emissions avoided that we identified were consistent with findings of a systematic review of 12 studies of CO_2_ emissions reduced per virtual care visit from various locations in North America and Europe before the pandemic.^22^ Among these studies, there was a correlation (*r* = 0.997) between distance traveled and kilograms of CO_2_ emissions avoided, suggesting that these estimates were consistent.^22^ Using their derived regression formula, we estimated that the mean trip length would be associated with 8.5 kg CO_2_ emissions avoided, which were within the estimates of this study. It is important to acknowledge that the provision of virtual care was also associated with a small amount of CO_2_ emissions. Although virtual care includes telephone calls and videoconferencing,^1^ 91% of virtual patient visits in Ontario were carried out over the telephone.^6^ Energy consumption for videoconferencing represented 1 of 10 000 of the CO_2_ emissions compared with emissions from travel in a prepandemic Canadian study.^23^ The CO_2_ emissions from cellular telephones or landlines are primarily related to phone production and infrastructure, which are not newly incurred with virtual care visits. Even including these costs, the CO_2_ emissions from a 10-minute cellular telephone call would account for only 1 g of CO_2_ emissions (compared with the 8.4-10.4 kg per virtual care visit in the present study).^24^

Patients avoided the costs of parking, public transit, and gasoline during virtual care visits. Such costs were estimated to be CAD $569 million (US $465 million) to CAD $733 million (US $599 million) during the study period. Although the median number of virtual care visits was low (and thus not likely to account for substantial monetary savings for many patients), among the 95th percentile of patients with more than 20 visits, this number may have been more apparent. Additional financial benefits were likely realized because of decreased time off from work because patients did not need to physically travel to the physician’s office to receive virtual care. In some cases, if there was flexibility in the workplace, it may have been possible for the virtual care episode to occur without any formal time off from work. This situation would be particularly relevant for parents of children who attended 5.2 million virtual visits and for working adults (aged 18-65 years) who had 41 million virtual visits.

We examined how the number of virtual care visits and the total travel distance avoided varied among patients on the basis of age, SES quintile, CCI score, and rural area of residence. The density plots (Figure 3 and Figure 4) better illustrate visible differences. The number of virtual care visits was greater among those older than 65 years and those with more comorbidities or living in an urban area. When we examined the travel distance avoided (which was proportional to the outcomes of CO_2_ emissions and gasoline costs), this number was also highest among those older than 65 years and those with more comorbidities. However, urban residents (who had more virtual care visits) had less travel distance avoided. This finding suggests that virtual care, although used less, offered more potential environmental benefits and patient cost savings for rural residents. A review of virtual care use among Canadians with cardiac conditions living in rural areas demonstrated reduced hospitalizations, better quality of life, and increased cost-effectiveness for patients and hospitals.^25^ Similarly, findings of the present study suggest that virtual care may help address some of the rural-urban health inequities by reducing patient travel costs, particularly at the extremes. For example, the 95th percentile of patients in rural areas realized greater than 2577 km of travel distance avoided compared with greater than 1191 km of travel distance avoided among the 95th percentile of urban residents (Table).

This research has implications for policy makers, physicians, and patients. It seems certain that virtual care will continue to be a part of medical practice.^1^ System-level investments in technologies that enhance virtual care, such as increased implementation of video-based services, may be partially justified on the basis of environmental and patient costs that can be avoided with virtual care. When physicians are considering offering virtual care, patients who are older, have more comorbidities, and are living in rural areas particularly may benefit by not incurring parking and other transportation costs, and offering virtual care when feasible may offer substantial environmental benefits in these patient groups. This finding is particularly relevant given that health care professionals are being asked to make sustainable health care planning and environmental care part of their ethical obligations.^26,27^ In addition, virtual care use was associated with direct financial benefit to patients, and it played a role in reducing their personal CO_2_ emissions. However, these benefits should not be interpreted as a reason to abandon in-person care, which is still necessary for many patient visits.

### Strengths and Limitations

The strengths and limitations of this study should be acknowledged. A study strength is the measurement of virtual care activity in a publicly funded Canadian health care system with minimal fiscal disincentives or regulatory limitations. We used a longer study period to include lulls and surges in COVID-19 cases. The estimates of orthodromic travel distance were based on postal code data and were expected to be accurate; however, they were likely conservative estimates given that previous studies showed the estimates needed to be corrected by a factor of 1.4 to account for the actual nonlinear road distance traveled.^28^

The models for CO_2_ emissions and patient expenses avoided should be interpreted as estimates rather than calculated values because factors, such as the use of public transit and the cost of parking, could not be reliably ascertained. To account for this variable, we used conservative assumptions and presented multiple scenarios to better show the range of possible values. We could not account for certain transportation mechanisms, such as walking, cycling, or air travel, although these methods would be expected to represent a small portion of patient travel. We assumed patients traveled from their area of residence to the physicians’ place of practice and then traveled back to their homes, but some people may have traveled to or from other locations. We may have overestimated the total travel distance avoided because of multidisciplinary clinics, which would normally allow a patient to see multiple physicians on the same day; however, in the data set, this type of visit appeared to be rare (0.05% of virtual visits were attributed to patients meeting with different physicians from the same institution on the same day). Similarly, there may have been some excess physician visits that would not have occurred if in-person visits were the only option, but previous research suggested this situation was not common during the pandemic.^6^ Furthermore, Ontario’s unique distributed geography, population density, and high adoption of virtual care may not be generalizable to other regions.

## Conclusions

This cross-sectional study found that virtual care during the first 22 months of the COVID-19 pandemic in Ontario, Canada, was associated with a large amount of CO_2_ emissions avoided due to reduced patient travel and with millions of dollars saved in parking, public transit, and gasoline costs. These benefits are likely to continue as virtual care is maintained as part of the health care system.
